# Exposure to the Dietary Supplement N-Acetyl-L-Cysteine during Pregnancy Reduces Cyclophosphamide Teratogenesis in ICR Mice

**Published:** 2018-10-18

**Authors:** Brittany M. Miller, Kyle K. Wells, Charles B. Wells, Xuan T. Lam, Marah E. Carney, Douglas S. Kepko, Morgan L. Mueller, Ronald D. Hood, Melissa M. Bailey

**Affiliations:** 1 Department of Biological Sciences, Emporia State University, Emporia, Kansas, USA; 2 Ronald D. Hood & Associates, Ponte Vedra, Florida, USA

**Keywords:** n-acetyl-l-cysteine, Cyclophosphamide, Mice, Teratogenesis, Thiol

## Abstract

Cyclophosphamide (CP) is a complex multifaceted developmental toxicant, with mechanisms of teratogenesis thought to include production of excessive reactive oxygen species (ROS). N-acetyl-L-cysteine (NAC) is a powerful antioxidant that may decrease the toxicity of certain anticancer drugs, such as doxorubicin and CP. The current study explored the potential of NAC to attenuate CP-induced damage to the conceptus. Mated ICR mice were orally dosed with 150 mg/kg/d NAC, 150 mg/kg/d NAC + 20 mg/kg CP, CP only, or vehicle only. CP was administered by intraperitoneal injection on gestation day (GD) 10, and NAC was given by gavage on gestation days 6–13. Dams were sacrificed on GD 17, and their litters were examined for adverse effects. There were significant reductions in the incidences of digit, limb, and tail defects, as well as anasarca and macroglossia, in fetuses exposed to the combination of NAC and CP, compared to fetuses exposed to CP only. NAC did not increase the incidence of any defects when compared to control. Fetuses exposed to NAC weighed significantly more than the average vehicle control fetus. The data indicate that NAC, a well-tolerated, relatively inexpensive antioxidant, appears to reduce the incidence of specific cyclophosphamide-induced malformations when administered prior to, concurrently with, and after exposure to CP.

## Introduction

Excessive oxidative stress is a concern during pregnancy, and physiological abnormalities leading to *in utero* death or birth defects induced by reactive oxygen species (ROS) are well known. Exposure to excessive amounts of oxidative stress can be caused by maternal treatment with drugs, such as cyclophosphamide (CP), or from a maternal disease state, such as diabetes [1,2].

CP is widely used to treat neoplastic and autoimmune diseases, including lymphoma, leukemias, lupus, multiple sclerosis, myasthenia gravis, scleroderma, and rheumatoid arthritis [3–5]. It is also one of the best known and studied proteratogens, causing a variety of birth defects, mainly central nervous system and skeletal abnormalities, in the fetuses of pregnant animals treated with the drug at dosages that are not observed to be maternally toxic [6–9]. Its teratogenic effects are thought to result from its bioactivation and breakdown, resulting in the production of phosphoramide mustard and acrolein. Phosphoramide mustard, acrolein, and the intermediate metabolite, 4-hydroperoxycyclophosphamidehave been shown to be teratogenic [6,10,11]. Side effects, such as hemorrhagic cystitis and hematuria, are common during CP therapy and are attributed to acrolein [9]. These side effects are greatly reduced by thiol compounds, such as 2-mercaptoethane sulfonate, which interacts with acrolein via a Michael addition reaction to “neutralize” the compound without compromising CPs anticancer efficacy.

ROS are key influencers in signaling pathways involved in proliferation, differentiation, and cellular fate during normal development [2]. In excess, however, ROS cause an imbalance between pro- and anti-oxidative species, leading to the condition known as oxidative stress. Cells accumulate ROS during the process of generating energy (ATP), as well as during processes arising from exogenous xenobiotic exposure, such as drug detoxification. ROS can bind covalently to DNA, protein, and lipid structures. This process of binding, i.e., oxidative stress, alters cellular function and can result in *in utero* death [12]. Thus, the teratogenic effect of CP is believed to come at least partially from its ability to induce oxidative stress within the system, and from its ability to deplete glutathione (GSH), although its major mechanism is generally thought to be the induction of DNA crosslinking and strand breakage [11,13,14].

The adverse effects caused by excessive ROS can be balanced with antioxidants, which are effective *in vitro* for preventing conditions associated with oxidative damage, through free radical scavenging [2,15,16]. Antioxidants work mainly by donating an electron to stabilize ROS [17]. Some of these antioxidants, e.g., glutathione and melatonin, are produced in the body, while many others, e.g., vitamins C and E are obtained from dietary supplements or food [18]. This laboratory has demonstrated that the antioxidants in green tea extract (epigallocatechingallate, among others) can significantly reduce specific CP-induced birth defects [19].

N-acetyl-L-cysteine (NAC) is a thiol-containing cysteine derivative that was introduced as a mucolytic agent in the 1960s and is used therapeutically to treat acetaminophen overdoses [20–22]. This well-known thiol antioxidant can function as both a redox buffer and a free-radical scavenger against endogenous free radicals or xenobiotics, both *in vitro* and *in vivo* [23,24]. NAC offers protection from the toxicity of certain anticancer drugs, including doxorubicin and CP [20]. Following its uptake, NAC is deacetylated to yield L-cysteine, which stimulates intracellular glutathione (GSH) production [23]. GSH, a tripeptide made up of glutamic acid, glycine, and cysteine, plays a key role in protecting cells against toxicants, oxidants, and DNA damaging agents [20,25]. NAC also shows nucleophilic properties, which allow it to combat free radicals through the processes of conjugation and reduction [26].

The effects of single exposures to thiol compounds, including exogenous gluthatione, cysteine, and 2-mercaptoethane sulfonate (MESNA), on the teratogenicity of CP has been studied [27–30]. Although other studies have examined the interaction between NAC and CP, no published studies to date have addressed the potential for protective effects of subchronic exposure of NAC against CP teratogenesis in a mammalian model. The current project examined the effects of NAC on the *in utero* development of ICR mice, using CP to induce oxidative stress and DNA alkylation. Given the antioxidant properties of NAC, it was not unreasonable to believe that NAC might attenuate the negative effects on embryo-fetal development induced by prenatal exposure to CP.

## Materials and Methods

### Animals and husbandry

Male and female ICR mice were purchased from Harlan Laboratories (Indianapolis, IN, USA) and housed at Emporia State University’s USDA-approved animal facility. The animal facility was maintained at 22±3°C at 50–70% humidity, with a 12-hour light/dark cycle. The mice were kept in same-sex gang housing and acclimated for a minimum of two weeks prior to mating. Mice received Teklad LM-485 rodent chow from Harlan Laboratories (Madison, WI, USA) and tap water and were fedad libitum.

After the acclimation period, each mouse was uniquely identified by ear punch. The mice were bred naturally, two females to one male. Females were examined for evidence of a copulation plug three times (8am, noon, and 8pm) on the day of pairing and every day thereafter for one week. GD0 was defined as the day a copulation plug was found. Mated females were housed individually and randomly assigned to treatment groups. All procedures performed on mice were in accordance with established guidelines set by Emporia State University’s Institutional Animal Care and Use Committee (permit #ESU-ACUC-14-001).

### Test chemicals

All chemicals were purchased from Sigma-Aldrich (St. Louis, MO, USA). NAC solution was prepared at a concentration of 15 mg/ml in deionized (DI) H_2_O to deliver a dosage of 150 mg/kg. Cyclophosphamide solution was prepared at a concentration of 2 mg/ mL (delivering a dosage of 20 mg/kg) in normal saline solution. Every solution was prepared freshly on the day that it was administered.

### Treatments

Females were randomly assigned to one of the four treatment groups: vehicle control, DI H_2_O (n = 25); NAC, 150 mg/kg/d (n = 25); NAC, 150 mg/kg/d + CP 20 (n = 29), mg/kg/d; or CP 20 (n = 22), mg/ kg/d. Dams were weighed on GD 0 and prior to every dosing. NAC or DI H_2_O was administered by gavage from GD 6–13. CP or saline was administered by intraperitoneal injection on GD 10.

### Data collection

On GD 17, females were euthanized via CO_2_ overdose, their uteri were exposed, and the numbers of resorptions and dead or live fetuses were recorded. Each of the live fetuses was removed from the uterus, weighed, and examined for gross malformations. Maternal body weight, minus the weight of the gravid uterus, was then recorded to determine if there were differences in maternal weight gain among the treatment groups. Fetuses were initially fixed in 70% ethanol for preservation and then cleared and stained by the double staining technique described by Webb and Byrd [31].

### Data analysis

The litter was used as the experimental unit for statistical analyses. Mean fetal weight, mean maternal weight gain, and the incidence (as percentage of affected fetuses) of each defect were calculated. Gross defects were grouped for analysis as follows: head (exencephaly and encephalocele), digit (polydactyly, oligodactyly, syndactyly, brachydactyly, and combinations thereof), limb (meromelia, phocomelia, and talipes), tail (short or bent tail), macroglossia, anasarca, and ablepharia. Incidences of defects per litter, maternal weight gain, and mean fetal weight were analyzed by one-way analysis of variance (ANOVA), followed by an LSD post hoc test to determine specific differences among groups (*p* ≤ 0.05). Both ANOVA and LSD post hoc tests were calculated by use of the Statistical Package for the Social Sciences (SPSS), version 14.0 for Windows (SPSS Inc., Chicago, IL).

## Results

### Maternal data

There were no significant differences in maternal weight gain among the treatment groups (Table 1). No clinical signs of maternal toxicity, such as lethargy, ataxia, ocular discharge, nasal discharge, abnormal respiration, or piloerection, were noted among any of the treatment groups.

### Fetal data

**Figure 1:**
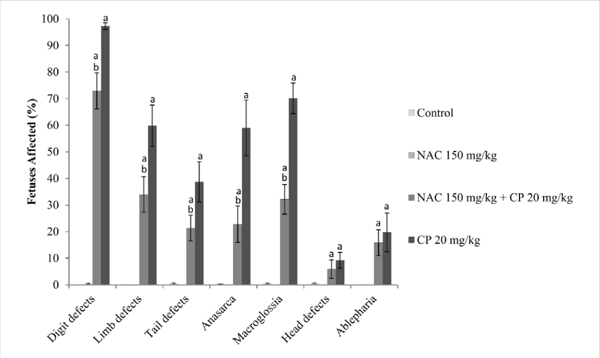
Incidence of Gross Malformations in Mouse Fetuses Following Maternal Exposure to Cyclophosphamide (CP) During Gestation, With or Without Exposure to N-acetyl-L-cysteine (NAC) aDiffers significantly from controls (p< 0.05)bDiffers significantly from 20 mg/kg/day CP (p< 0.05).

The numbers of resorbed or dead fetuses were not significantly different among any of the study groups. Fetal weight in the NAC-only group was significantly higher than the control (*p* = 0.024). Exposure to CP, either alone or in combination with NAC, significantly reduced fetal weight compared to the vehicle control value (*p* ≤ 0.01). Administration of NAC was associated with an apparent small increase in fetal weight compared to the weight of fetuses exposed to CP only, but the difference was not significant (*p* = 0.060) (Table 1). The percentage of fetuses displaying any type of gross malformation was not significantly different between the vehicle control group and those exposed to NAC only (Figure 1). The incidences of digit, limb, and tail defects were significantly reduced in the NAC + CP group compared to the CP group (*p* < 0.01). Anasarca and macroglossia were also significantly reduced in fetuses exposed to the combination of NAC and CP, compared to fetuses exposed to CP only (*p* < 0.01). There were no significant differences in head defects or ablepharia between combined NAC + CP and CP only groups.

**Figure 2:**
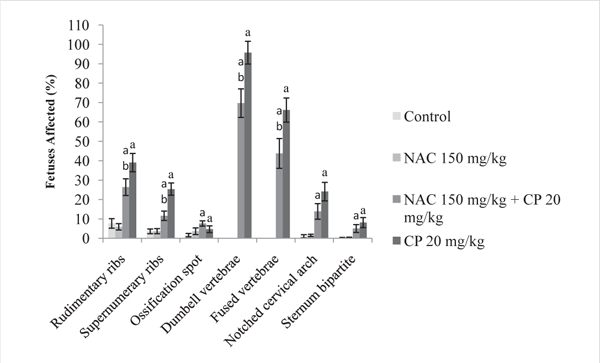
Incidence of Skeletal Variations and Malformations in Mouse Fetuses Following Maternal Exposure to Cyclophosphamide (CP) During Gestation, With or Without Exposure to N-acetyl-L-cysteine (NAC) ^a^ Differs significantly from controls (p< 0.05) ^b^Differs significantly from 20 mg/kg/day CP (p< 0.05)

The incidences of skeletal abnormalities, as with gross malformations, were not significantly different between the controls and the NAC-only treatment group (Figure 2). The percentages of fetuses with rib (rudimentary or supernumerary) variations and vertebral (dumbbell or fused centra) defects were significantly reduced in fetuses exposed to NAC and CP, in comparison with fetuses exposed to CP alone (*p* < 0.05). No statistical difference was seen in other vertebral (notched cervical vertebrae) or rib anomalies (ossification spots), or in skeletal malformations, such as bipartite sternum.

## Discussion

NAC is an effective chemopreventative agent and has shown many cancer-preventive effects [32,33]. The objective of this study was to examine the potential of NAC as a protective agent against the damage caused by theproteratogen, cyclophosphamide. The mechanism behind CP’s teratogenic properties is thought to be caused, at least in part, by the ability of CP to induce oxidative stress by flooding the system with ROS [14]. One way this damage can be balanced is through the use of antioxidants [2].

By itself, CP causes neither neoplastic nor teratogenic effects until it is bioactivated [7]. The bioactivation and metabolism of CP to 4-OHCP, aldophosphamide, and ultimately, the active metabolites phosphoramide mustard and acrolein and the inactive metabolites 4-ketoCP and carboxyphosphamide, have been elegantly reviewed elsewhere [7,9,34].

Glutathione (GSH) conjugation is the main phase II detoxification mechanism for CP. The key enzyme in this process is glutathiones-transferase (GST). NAC is a precursor to GSH. NAC becomes deacetylated to form 1-cysteine, which supports biosynthesis of GSH. When GSH levels are increased, so are GST levels. NAC not only increases the amount of GST but has also been shown to enhance its activity as well. The increase in GSH and GST caused by NAC has resulted in NAC being a crucial component in the treatment of acetaminophen overdose [21,22]. NAC has also been used to treat GSH deficiency in infections (including HIV) and genetic disorders [3,5]. Thus, NAC’s support of phase II metabolism is one of the likely mechanisms for reducing the teratogenic effects of CP observed in the current study. There is strong evidence to suggest that the protective effects of NAC are likely maternal, rather than fetal, as bioactivation of CP is thought to take place in the maternal liver [6].

In addition to its potential influence on phase II metabolism, NAC is a well-known antioxidant that is very efficient as a redox buffer and as a free radical scavenger, because of its thiol group [23,32]. NAC is thought to work by two possible mechanisms. As discussed above, NAC is a precursor to GSH [33]. NAC also can act as a strong nucleophile, attacking oxidant radicals directly. The nucleophilic and reducing properties of NAC alter mutagenicity of direct-acting compounds, such as epichlorohydrin, sodium dichromate, and hydrogen peroxide, and of promutagens by reducing chromatid breakage. By protecting nuclear enzymes and correcting DNA methylation, NAC also regulates DNA repair after damage has already been done [20,32].

The findings in this study are comparable to those of other studies that have examined the beneficial effects of NAC. Botta et al. [35] studied several agents in an effort to prevent cyclophosphamide-induced cystitis. Of all the agents in their study, NAC was the most effective. A later study by Moradi et al. [5] found that NAC reduced the formation of proinflammatory cytokines, such as interlukin-8 and tumor necrosis factor. Another proposed mechanism was that NAC was inactivating alkylating metabolites of CP [35]. The notion that NAC acts at least in part by neutralizing free radicals was further supported when Doroshow et al. [36] examined the effect of NAC on doxorubicin toxicity in mice. NAC was effective in blocking cardiac toxicity, but it did not affect uptake or metabolism of doxorubicin in the spleen or the liver. One proposed reason as to why it did not affect uptake or metabolism is because doxorubicin’s major cytotoxic effect on tumor cells is not related to the formation of free radicals.

The dosage of NAC (150 mg/kg/d) to which the dams were exposed in the current study would be considered pharmacological in comparison with the suggested intake for use of NAC as a dietary supplement. According to label information on readily available NAC supplements, the suggested dosage for use of NAC as a dietary supplement is typically 600–1200 mg per day (approximately 8–17mg/ kg for a 70 kg adult).

The exact mechanism for the results observed in this study remains unclear; however, previous literature strongly suggests that NAC was acting by supportingphase II biotransformation by causing an increase in GST, by directly scavenging and neutralizing free radicals created by CP, or by some combination of the two. The data from this study show that exposure to NAC at pharmacological dosages, could reduce the incidence and severity of birth defects caused by CP exposure in an animal model, while NAC itself did not cause maternal toxicity or embryotoxicity. While the results of this study are unlikely to change therapeutic recommendations of CP use during pregnancy, they do suggest that the use of NAC supplementation during pregnancy may be an avenue worth pursuing, given the affordability, ease of use, and popularity of NAC as a dietary supplement, particularly in women with conditions or exposures that may lead to the production of excess ROS. However, the implications for human pregnancies cannot be directly extrapolated from the results of this study alone.

## Figures and Tables

**Table 1:** Maternal and Litter Parameters of Mice Treated During Gestation With Cyclophosphamide (CP) With or Without Exposure to N-acetyl-L- cysteine (NAC).

	Treatment and Dose (mg/kg/day)			
	Vehicle Control	NAC 150	NAC150 + CP 20	CP 20
Litters examined (Fetuses/Litters)	349/25	353/25	378/29	173/22
Maternal weight gain (g ± SEM)	12.98 ± 0.58	14.21 ± 0.54	12.62 ± 0.59	13.44 ± 0.47
Fetal weight (g ± SEM)	1.00 ± 0.02	1.07 ± 0.02^a^	0.71 ± 0.02^b^	0.65 ± 0.02^b^
Implantations (mean ± SEM)	14.24 ± 0.39	14.28 ± 0.45	13.70 ± 0.37	13.71 ± 0.37
Resorbed dead fetuses (% ± SEM)	1.60 ± 0.59	1.08 ± 0.63	5.13 ± 3.17	9.38 ± 4.84
Litters with resorbed or dead fetuses (No./%)	5/20	3/12	6/21	7/32

a Differs significantly from vehicle control (*p* ≤0.05)

b Differssignificantly from vehicle and NAC controls (*p*< 0.01).
